# Using flattening filter free beams in electronic tissue compensation whole breast irradiation with deep inspiration breath hold

**DOI:** 10.1002/acm2.13109

**Published:** 2020-12-03

**Authors:** Sarah B. Wisnoskie, Xiaoying Liang, Andrew O. Wahl, Nathan R. Bennion, Andrew D. Granatowicz, Sumin Zhou, Dandan Zheng

**Affiliations:** ^1^ Department of Radiation Oncology University of Nebraska Medical Center Omaha NE USA; ^2^ Department of Radiation Oncology University of Florida Proton Institute Jacksonville FL USA

**Keywords:** breast cancer, breath hold, DIBH, ECOMP, external beam, FFF

## Abstract

**Purpose:**

In order to reduce heart dose, DIBH has become a common practice in left‐sided whole breast irradiation. This technique involves a significant strain on patients due to the breath‐hold requirements. We hereby investigate the dosimetric and delivery feasibility of using flattening filter free (FFF) energies with electronic tissue compensation (ECOMP) planning technique to reduce the required breath‐hold lengths and increase patient compatibility.

**Methods:**

Fifteen left‐sided, postlumpectomy patients previously receiving DIBH whole‐breast radiotherapy (266cGy x 16fx) were retrospectively planned using ECOMP for both 6X and 6X‐FFF. A dosimetric comparison was made between the two plans for each patient using various dosimetric constraints. Delivery feasibility was analyzed by recalculating the 6X ECOMP plan with 6X‐FFF without replanning (6X‐FFF QA) and delivering both plans for a one‐to‐one comparison using Gamma analysis. Beam‐on times for the 6X and 6X‐FFF plans were measured. For all tests, Wilcoxon signed‐rank test was used with *P* < 0.05 as significant.

**Results:**

No statistical difference was observed between 6X and 6X‐FFF plans for most dosimetric endpoints except contralateral breast D_max_ (*P* = 0.0008) and skin D_max_ (p = 0.03) and D_min_ (*P* = 0.01) for which 6X‐FFF showed favorable results when compared with 6X. 6X‐FFF significantly reduced beam‐on times for all patients by 22%–42% (average 32%). All plan QAs passed departmental gamma criteria (10% low‐dose threshold, 3%/3mm, >95% passing).

**Conclusion:**

ECOMP planning with FFF was found feasible for left‐sided breast patients with DIBH. Plan quality is comparable, if not better, than plans using flattened beams. FFF ECOMP could significantly reduce beam‐on time and required breath‐hold lengths thereby increasing patient compatibility for this treatment while offering satisfactory plan quality and delivery accuracy.

## 1 Introduction

Breast cancer is the most diagnosed form of noncutaneous cancer in American women and results in the second highest number of cancer‐related deaths behind only lung cancer. It was estimated that in the United States, one in eight women will be diagnosed with breast cancer at some point in their lifetimes.^1^ Radiotherapy plays an essential role in breast cancer treatment and is used for over half of all breast cancer patients.^2^ It has been shown to both decrease loco‐regional recurrence rates and improve overall survival for breast cancer patients.^3^ Due to the effective tumor control for these patients and the long‐term survival of most of them, one central theme of radiotherapy advances in the recent years has been to reduce the treatment toxicity and long‐term side effects.

In 2013, a seminal paper by Darby et al. showed that a non‐negligible risk of heart disease and coronary events is associated with breast cancer radiotherapy and the risk is estimated to increase 4–7% for each 1 Gy in mean heart dose.^4^ Enlightened by the study, clinicians have grown increasingly cautious about the cardiac risk. Special techniques have been more commonly employed in modern breast radiotherapy to minimize the radiation dose to the heart, especially for left breast cancer where heart dose tends to be higher due to the close proximity to the treatment targets. Above all, Deep Inspiration Breath Hold (DIBH) is the most popular technique used to reduce the heart dose in breast radiotherapy, especially with the increasing availability of the surface guidance technology. It increases the distance from the breast to the heart by having patients hold their breath for the duration of each treatment beam, which typically takes 20–50 s. DIBH has shown a reduction of dose to the heart between 31% and 80%.^5^, ^6^ While the benefits are undeniable, there are many patients who are not well suited for DIBH due to the physical demand it requires. Patients with chronic obstructive pulmonary disease (COPD) or many other respiratory diseases often cannot perform the breath hold of the required length and are hence unable to use the DIBH technique. For patients that can, radiotherapy delivery uncertainty could also increase with prolonged treatment time due to possible drifts at the end of a long breath hold and other patient motions.

Therefore, in this study we investigate whether flattening filter free (FFF) beam mode could be utilized to reduce the required breath‐hold lengths for DIBH breast radiotherapy, making the technique more widely applicable and more accurate by decreasing delivery time thereby decreasing motion uncertainties. While flattened photon beams with flattening filters are conventionally used for radiotherapy, FFF beams have become available on regular LINACs within the past decade. Much higher dose rates are available for FFF beams than the flattened beams. For example, the dose rate is up to 2400 MU/min for 10X‐FFF beam and 1400 MU/min for 6X‐FFF mode compared with the 600 MU/min maximum dose rate on the flattened photon energies on a TrueBeam (Varian Medical Systems, Palo Alto, CA, USA). Taking advantage of the high dose rates, FFF beams are primarily used to reduce the treatment time in stereotactic treatments where high doses are treated each fraction. LINAC‐based stereotactic treatment planning with FFF beams is straightforward because most cases use inverse planning. Even in forward‐planned cases with conical or conformal beams or arcs, the target is very small and the allowed target dose nonuniformity is large, therefore the relatively flat central portion of FFF beam profile does not complicate the planning.

In contrast, external beam breast radiotherapy has a very large target size and a strict target dose uniformity requirement (commonly only up to 7% hot). Conventional 3D conformal forward planning with tangential fields is therefore challenging to achieve with FFF beams. However, a recently available planning technique, electronic tissue compensation (ECOMP), could easily employ FFF beams. In ECOMP, the planner creates and manually optimizes fluences (or beam intensity) instead of beam apertures, and the plan is then delivered via a sliding window technique. The ECOMP planning technique has been gaining popularity as it is efficient and has been shown to increase target dose uniformity.^7^, ^8^, ^9^ Since in ECOMP planning the planner operates on fluences instead of beam apertures, it makes a perfectly feasible planning technique to test FFF beams for DIBH breast radiotherapy. In this work, we report a designed experiment to test the dosimetric and delivery feasibility of FFF ECOMP plans, in order to reduce required breath‐hold lengths and increase patient compatibility of DIBH breast radiotherapy while not sacrificing plan quality.

## 2 Materials and Methods

### 2.1 Patient selection and contour delineation

Under the approval of the Institution Review Board, 15 left‐sided postlumpectomy patients with separations of less than 24 cm treated with 6X whole breast irradiation at the University of Nebraska Medical Center between 2017 and 2020 were randomly selected for this retrospective study. Breast tissue was identified at the time of simulation by palpation and marked with wire for delineation on the simulation computed tomography (CT) scan. The scan was acquired using a Somatom Definition AS CT scanner (Siemens Medical Solutions, Forchheim, Germany) in 3 mm slice thickness, with the patients lying supine on a breast board with both arms up.

Target contours were drawn by the attending radiation oncologist according to our departmental protocol, including breast PTV (determined by wire placed at time of simulation), evaluation PTV or PTVe (breast PTV minus 5 mm skin), Lumpectomy GTV (lumpectomy cavity), Lumpectomy PTV (1.7 cm margin around GTV), and Lumpectomy evaluation PTVe (Lumpectomy PTV minus 5 mm skin). OARs include heart, ipsilateral lung, contralateral breast, and skin (defined as the 5 mm inner wall from the external body contour that overlaps with the breast PTV contour).

### 2.2 Treatment planning

**Fig 1:**
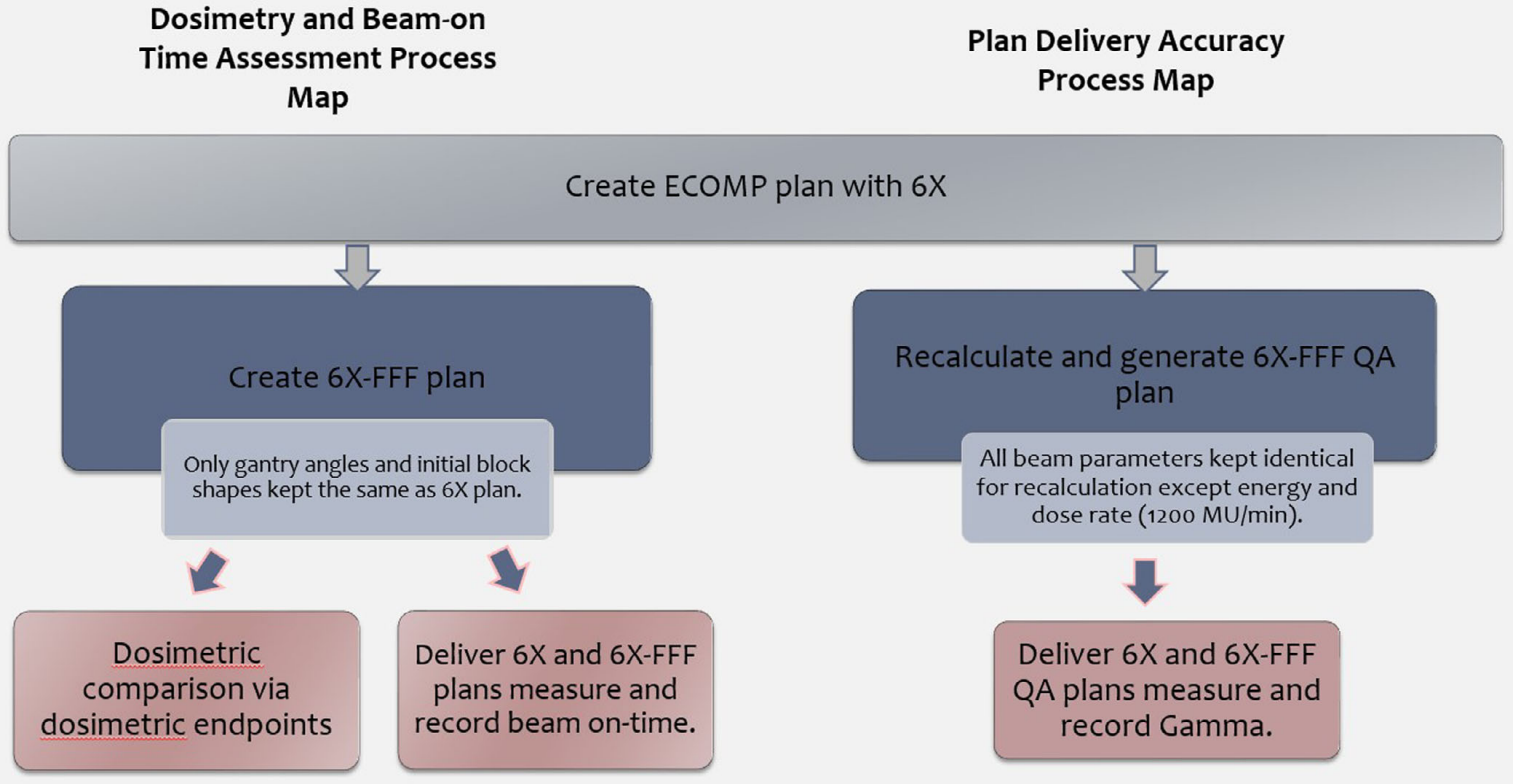
Process flow chart.

The plans for this study were retrospectively created using the ECOMP planning technique in Eclipse v.15 (Varian Medical Systems, Palo Alto, CA, USA). A process map of the study is shown in Fig. 1. For the dosimetric assessment, two parallel plans were created for each patient, one using 6X and the other using 6X‐FFF of a TrueBeam linear accelerator (Varian Medical Systems, Palo Alto, CA, USA). On each case, the two plans used identical beam angles (two tangential fields), field sizes (including 2cm flash), isocenter, and aperture shapes determined by the attending physician. For ECOMP planning, a block was drawn copying the aperture shape so that when the new irregular surface compensator was added and the fluence map was created, the block can be used to erase fluence outside the block area. Patients were planned to a prescribed dose of 2.66 Gy × 16 (42.56 Gy) to the PTVe. The plans were normalized to maximize the dose to lumpectomy GTV while still keeping a hotspot of < 107%. The penetration depth was defined as 50% depth. This identifies the percent depth at which a uniform dose will be delivered for the two tangent beams. The fluence map generated was then modified by the planner to reduce hotspots and boost cold spots.

### 2.3 Plan analysis and dosimetric assessment

To evaluate the plan quality using 6X vs 6X‐FFF, dosimetric comparison was performed between the two parallel plans on each case using our institutional criteria for postlumpectomy whole breast irradiation as the dosimetric endpoints. The dose endpoints and ideal planning objectives are shown in Table 1 for both the targets and OARs. Note that we added skin D_max_, D_min_, and D_mean_ for the purpose of this study although our institution does not currently consider skin constraints in the planning process.

**Table 1:** Dosimetric ideal planning objective and endpoint comparison (mean ± 1 standard deviation) for 6X and 6X‐FFF ECOMP plans including Wilcoxon signed‐rank test p‐values (bold values indicate statistical significance).

Structure	Endpoint (units)	Ideal Objective	6X	6X‐FFF	*p*‐value
PTVe	V95% (%)	>95%	97.3 ± 1.3	97.4 ± 1.2	0.61
	V105% (%)	<10%	2.9 ± 3.1	3.4 ± 2.6	0.78
	D_max_ (%)	<107%	106.2 ± 0.9	106.4 ± 0.7	0.87
Lump PTVe	V95% (%)	>95%	99.9 ± 0.2	99.9 ± 0.2	n/a
Lump GTV	D_min_ (%)	>100%	99.3 ± 4.1	99.5 ± 3.1	0.91
Heart	D_mean_ (cGy)	<250 cGy	76.4 ± 19.8	74.2 ± 17.9	0.12
	D_max_ (cGy)	<3500 cGy	1388.8 ± 875.3	1264.4 ± 776.6	0.5
Ipsilateral Lung	V16Gy (%)	<15%	8.8 ± 3.6	8.8 ± 3.3	0.82
Contralateral Breast	D_max_ (cGy)	<300 cGy	302.5 ± 270.2	233.8 ± 229.4	**0.0008**
Skin	D_mean_ (cGy)	N/A	3525.9 ± 35.9	3519.6 ± 29.8	0.31
	D_max_ (cGy)	N/A	4469.8 ± 38.4	4441.5 ± 58.3	**0.03**
	D_min_ (cGy)	N/A	762.3 ± 146.5	790.3 ± 170.6	**0.01**

### 2.4 Plan delivery assessment

To evaluate the delivery time, both 6X and 6X‐FFF plans were delivered and the beam‐on time was recorded. A dose rate of 600 MU/min was used for 6X and that of 1200 MU/min was used for 6X‐FFF plans.

In addition, to assess plan delivery accuracy a 6X‐FFF QA plan was created and QA gamma passing rates were compared between the 6X and the corresponding 6X‐FFF QA plans. To create the 6X‐FFF QA plan, the 6X plan was copied and the energy changed to 6X‐FFF with the corresponding changed dose rate while keeping all other plan parameters (MUs, field weights, fluence, etc.) constant. Despite keeping all the parameters the same, the leaf sequence must be recalculated when energy and dose rate are changed which results in a slightly different leaf sequence between the 6X and 6X‐FFF QA plans. The decision to create the 6X‐FFF QA plan for evaluation, rather than using the 6X‐FFF plans created for dosimetric assessment, is in an effort to make QA comparison between the two energies more objective. The QA analysis was done using the gamma passing rate (3%/3 mm, 10% low‐dose threshold) with portal dosimetry (Varian Medical Systems, Palo Alto, CA, USA).

### 2.5 Statistical analysis

The dosimetric endpoints, QA gamma passing rates, and delivery beam‐on time were compared between the two energy groups using a Wilcoxon signed‐rank test, with *P* < 0.05 considered as significant.

## 3 Results

### 3.1 Dosimetric comparison

**Fig 2:**
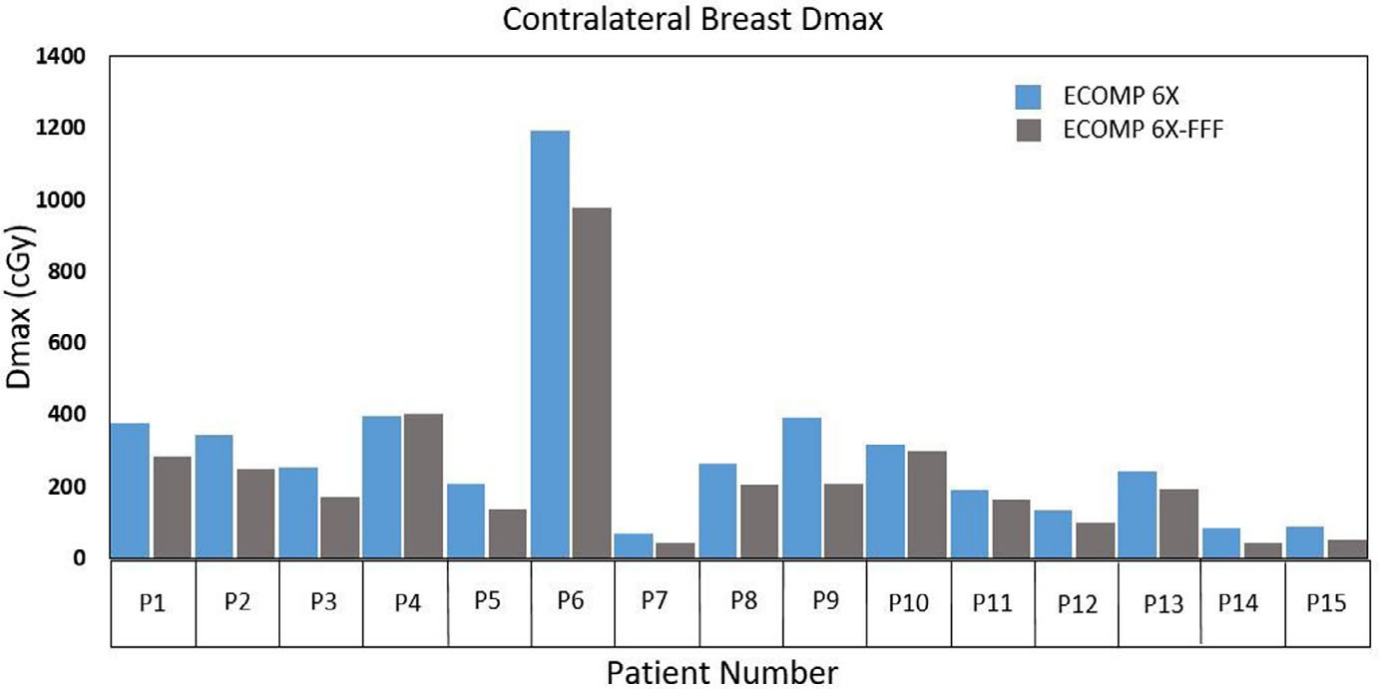
Contralateral breast D_max_ (cGy) comparison between 6X and 6X‐FFF for all patients.

Our patient cohort had a mean PTVe volume of 566.87 cc ± 225.47 cc and separation of 20.44 cm ± 2.53 cm. The dosimetric analysis results for each endpoint are shown by mean values and standard deviations in Table 1. For target coverage, no statistically significant difference was noted between the two plans. The mean differences are also very small and likely insignificant clinically. A *P*‐value for the lumpectomy PTVe was unable to be obtained since there was no difference in the V95% coverage between the 6X and 6X‐FFF plans. For OARs, statistically significant differences were only observed in the contralateral breast D_max_ and skin D_max_ and D_min_. For the contralateral breast, the D_max_ for 6X‐FFF showed a mean reduction of 68.7 cGy from 6X. Figure 2 shows the contralateral breast D_max_ for both 6X and 6X‐FFF for all patients. Note that while a couple of plans did not meet the ideal planning objective for contralateral breast, this is due to the use of deep tangents to cover internal mammary nodes for these patients in the original clinical plans which were deemed acceptable. The skin D_max_ for 6X‐FFF was slightly lower than 6X (by an average of 28.3 cGy) and the D_min_ for 6X‐FFF was slightly higher than 6X (by an average of 28 cGy), indicating a slightly more uniform skin dose when using 6X‐FFF. No significant differences were noted for the heart or ipsilateral lung. While not statistically significant, the heart had a reduction in D_max_ between 6X‐FFF and 6X (1264.4 cGy vs 1388.8 cGy, respectively).

**Fig 3:**
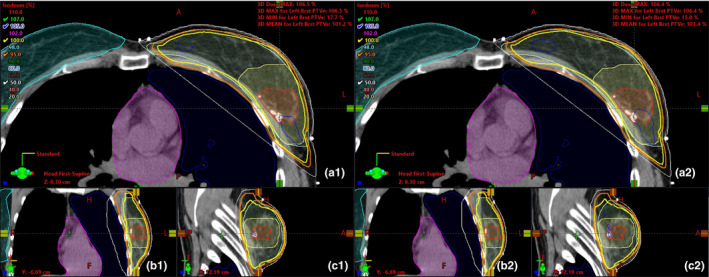
Plan comparison between ECOMP 6X (1) and ECOMP 6X‐FFF (2) in axial (a), coronal (b) and sagittal views (c). Contours shown include contralateral breast (cyan), heart (magenta), ipsilateral lung (dark blue), breast PTVe (light green), lumpectomy PTVe (yellow), and lumpectomy GTV (red) and are all displayed with segment color display.

**Fig 4:**
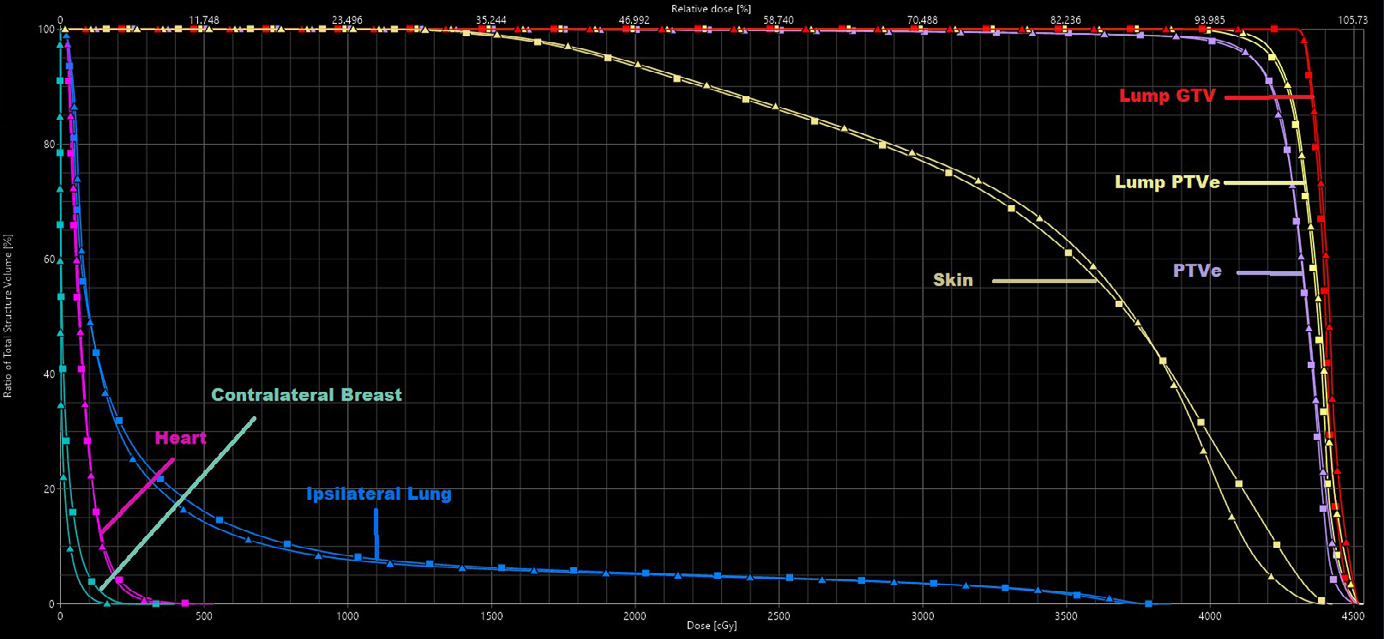
DVH comparison between 6X (square) and 6X‐FFF (triangle) plans.

The ECOMP plans of both energies for a representative patient are shown with isodose lines in axial, sagittal, and coronal views (Figure 3) and in the comparative dose–volume histogram (Figure 4). As can be seen, the two plans are similar with nearly identical target coverage and OAR doses except slightly lower contralateral breast and skin dose on the 6X‐FFF plan.

MUs for each plan as well as the difference between the 6X and 6X‐FFF plans are shown in Table 2. Higher MUs were needed in the 6X‐FFF plans due the peaked beam profile and softer beam energy.

**Table 2:** MUs per plan for 6X ECOMP and 6X‐FFF ECOMP.

Patient #	6X MUs	6X‐FFF MUs	% difference
1	418	559.2	33.78%
2	507.8	560	10.28%
3	452.3	552.7	22.20%
4	492.1	577.5	17.35%
5	441.4	633.6	43.54%
6	449.6	552.2	22.82%
7	435.7	637	46.20%
8	435.4	552.5	26.89%
9	512.3	533.4	4.12%
10	465.8	640	37.40%
11	444.4	570.5	28.38%
12	440.1	604.7	37.40%
13	522	768.1	47.15%
14	424.4	536.6	26.44%
15	445.1	602.2	35.30%

### 3.2 Delivery comparison

The QA gamma results are shown in Table 3. A p‐value for the difference in gamma could not be obtained since many patients had identical passing gamma results. All plans met our institutional criteria for passing QA (10% threshold, 3%/3 mm gamma < 1 for> 95% of points). In a few cases, the gamma passing rates for the 6X‐FFF plans were slightly lower than the 6X plans with the greatest difference being 2.9% on one case.

**Table 3:** Composite QA γ results for 6X and 6X‐FFF QA (3%/3 mm, 10% low‐dose threshold, γ < 1).

Patient #	Composite QA 6X (γ)	Composite QA 6X‐FFF (γ)
1	100	99.8
2	99.8	99.7
3	100	100
4	100	100
5	100	100
6	100	100
7	98.8	98.3
8	100	99.9
9	100	100
10	100	100
11	99.6	99.5
12	100	100
13	100	99.9
14	99	96.1
15	100	100

As expected, with a higher dose rate, the 6X‐FFF plans recorded shorter beam‐on time than the corresponding 6X plans. The percentage decrease in beam‐on time for the 6X‐FFF plan when compared to the 6X plan is shown in Table 4. The difference was statistically significant (*P* = 0.0006) with a mean decrease of 32%.

**Table 4:** Beam‐on time (delivery time) for both 6X and 6X‐FFF plans.

Patient #	6X delivery time (s)	6X‐FFF delivery time (s)	% time decrease
1	46.35	32.48	29.9
2	44.22	34.5	22.0
3	50.12	33.02	34.1
4	55.98	36.4	35.0
5	49.62	39.88	19.6
6	50.65	35.8	29.3
7	49.48	36.93	25.4
8	47.28	34.29	27.5
9	54.19	31.42	42.0
10	51.59	31.42	39.1
11	48.9	30.52	37.6
12	49.7	32.65	34.3
13	58.77	39.24	33.2
14	47.47	30.01	36.8
15	49.07	31.95	34.9

## 4 Discussion

Traditionally, whole breast or chest wall irradiation has been treated with two tangential fields, which has superior delivery robustness compared with IMRT plans with alternative beam arrangements yet often shows an equivalent dosimetry. With the tangential beam arrangement, 3D planning techniques have evolved over time from standard 3D with wedges to field‐in‐field (FiF), and most recently, to ECOMP. Compared with FiF, ECOMP has been shown to increase homogeneity of the PTV and decrease V20Gy of the ipsilateral lung.^7^, ^8^, ^9^ Additionally, ECOMP plans were found to require slightly higher MUs.^10^

In this study, all the ECOMP plans created for the study were dosimetrically equivalent to, or better than, their clinical FiF counterparts on all endpoints. Generally, ECOMP plans require less planning time than their FiF counterparts although FFF ECOMP plans can take slightly longer to plan than flattened ECOMP plans due to the added complexity as a result of their peaked profiles. This difference in planning time is usually negligible. It should also be noted that because a sliding window MLC modulation is used in ECOMP delivery, some institutions, including ours, perform an IMRT QA on all ECOMP plans. Other institutions may not require a patient‐specific QA for these plans because ECOMP is considered forward planning. Nevertheless, ECOMP breast planning has been gaining popularity due to improved planning efficiency and plan quality, and is routinely used in many clinics including ours. In our study, using the ECOMP technique, we analyzed plan equivalence, delivery accuracy, and delivery efficiency of 6X vs 6X‐FFF plans to assess whether it is feasible to use FFF beams to reduce the required breath‐hold lengths and improve the compatibility of DIBH tangential breast RT.

FFF beams have been used historically for SRS and SBRT. The use of FFF beams allows a dose rate escalation due to the removal of the flattening filter while sacrificing the flat profile which can be corrected for in the plan itself. Additionally, the peaked profile has a sharper penumbra which, while favorable for SRS/SBRT, can cause inhomogeneity when unaccounted for in conventional RT planning. Most often, plans using FFF beams use inverse planning which allows the treatment planning system to account for the lack of profile flatness in the optimization of the plan. For breast planning, the use of FFF beams has thus far been limited to specialized RT systems with only FFF beams. IMRT plans using the helical TomoTherapy system (Accuray Incorporated, Sunnyvale, CA, USA) have been shown to provide superior dose conformity and homogeneity which mostly comes from the inverse planning.^11^ On the more recently available Halcyon system (Varian Medical Systems, Palo Alto, CA, USA), the FFF plans were also shown to be comparable to the flattened plans on conventional LINACs.^12^, ^13^, ^14^ but showed an increase in superficial dose of 10%.^15^ Outside of these specialized systems, there have been very few studies on the application of FFF beams in breast RT. The only other study in the setting of breath‐hold breast RT to reduce beam‐on time investigated only IMRT techniques (tangential IMRT and VMAT with limited tangential arcs).^8^ In another study comparing different techniques for general breast treatment planning, ECOMP plans were shown to have within 3% mean target volume metrics and comparable OAR doses compared with wedged tangential, IMRT, and hybrid IMRT plans.^10^ Additionally, the increase in dose rate could cause concern about possible increased toxicity. While there are few reports discussing standard C‐arm linac FFF toxicities for breast radiotherapy, studies of O‐ring linacs have shown acceptable toxicities.^15^ Also, while not widespread, breast SBRT is used with acceptable toxicities.^16^ Compared with conventional 3D planning techniques, ECOMP provides a unique opportunity to utilize FFF beams for reducing beam‐on time and hence the required breath‐hold length in DIBH treatments.

In our study of 15 DIBH left‐breast patients with the ECOMP technique, all of the target coverage and hotspot endpoints had mean results within 1% between the 6X and 6X‐FFF plans and none of the differences were statistically significant. This indicates that the plans can be considered dosimetrically equivalent with regard to target coverage. As for the OARs, statistically significant differences were only observed for contralateral breast D_max_, and skin D_max_ and D_min_, all of which showed the 6X‐FFF to be favorable to the 6X plans. For contralateral breast D_max_, the 6X‐FFF plans had an average value of 233.8 cGy vs 302.5 cGy for the 6X plans. This could have potential clinical significance as women and especially younger women were found to have dose‐dependent higher risk of developing a secondary primary with increasing dose to the contralateral breast.^17^ For skin dose, 6X‐FFF plans showed a higher average D_min_ and a lower average D_max_, indicating a more homogeneous dose distribution in the skin than 6X plans, although the differences were small and on the order of 20–30 cGy. Since the primary concern about skin dose in whole breast irradiation is adequate dose to reduce the chance for tumor recurrence in the skin while also limiting radiation induced skin toxicities, the more homogeneous skin dose offered by FFF beams may be preferred. We speculate that the observed contralateral breast and skin dose differences on the 6X‐FFF plans were caused by the softer beam energy at the periphery of the large tangential fields. It is worth noting that another recent study comparing superficial dose of FFF plans on Halcyon and FF plans on TrueBeam with OSLD measurements on phantoms showed a 10% increase in the FFF plans.^15^ The varying results between our and their study could have come from the endpoint difference (OSLD measurements likely assess the D_mean_), MLC and beam difference in their study (Halcyon FFF vs TrueBeam FF), and the superficial dose calculation uncertainty of the commercial treatment planning system.

Similar to previous studies comparing FFF and FF beams for delivery efficiency that showed a reduction in delivery time between 18% and 39%,^10^, ^12^, ^14^, ^15^, ^18^ in our study the delivery time was significantly reduced for 6X‐FFF plans with a 32% average decrease. This difference could substantially reduce the strain of the DIBH on patients and make this heart‐sparing treatment technique more compatible in breast cancer patients to reduce the cardiac risk from the breast radiotherapy. This average decrease does consider the resulting MU increase in the 6X‐FFF plans (about 29% or 132 MUs). This increase in MU is necessary in order to maintain homogeneous dose delivery to the whole breast due to the beam profile of FFF beams since the profile is no longer flat but peaked at the center. It needs noting that this MU increase could also lead to an increased integral dose to the patient the toxicity of which needs to be investigated further. Regarding delivery accuracy, our QA tests showed excellent gamma passing results on the 6X‐FFF plans with the lowest passing rate at 96.1% and majority of the plans at or close to 100%, despite a slight decrease in gamma passing rates on some plans compared with their 6X counterparts. This indicates that despite the increased dose rate, FFF ECOMP plans could be deployed with acceptable delivery accuracy to reduce the respiratory strain on breast cancer patients to benefit from the cardiac risk reduction provided by DIBH breast irradiation. One limitation of our study is that due to the required leaf sequence recalculation when switching energies and dose rates, the QA comparison was not perfectly objective. Despite this, because all other parameters (MUs, field weights, fluence, etc.) were kept constant, the 6X‐FFF QA plans were considered to be a much closer analogue to the 6X plan than the 6X‐FFF plan for the delivery accuracy comparison.

Because both FFF beams and the ECOMP planning technique are gaining popularity in the clinic, there have been some similar studies to ours that compared FFF and flattened beams for breast planning.^10^, ^14^ However, despite the findings from these publications that FFF had comparable plan quality and a reduced delivery time than their flattened counterparts, FFF plans are very rarely used in the current clinical practice of whole breast irradiation. Likely contributing to this lack of clinical application may be the lack of a necessity for shortened delivery time in the general breast RT setting and the concerns against IMRT which has often been the focus of these earlier FFF breast investigations. Due to clinical consideration of respiratory motion interplay and practical issue of insurance approval, tangential plans still by and large dominate breast RT. The recent rapid rise of heart‐sparing left‐breast RT with DIBH presented a patient population for whom FFF ECOMP plan could be uniquely beneficial while free of the above‐mentioned concerns. We therefore chose to focus on comprehensively addressing the dosimetry, delivery efficiency as well as delivery accuracy aspects of this specific application to demonstrate the applicability of FFF ECOMP breast treatment with DIBH on C‐arm linacs. While the individual technological components in our study may not be novel, the application is unique and clinically meaningful.

This study is not without limitations. In our study we selected 15 left‐sided DIBH whole breast irradiation patients. Although the involved radiation physics and hence dosimetry seem similar, our results and conclusion may not be directly generalized to chest wall irradiation for postmastectomy patients or for DIBH treatments on right‐sided breast cancer. Furthermore, we only investigated the most common 6X energy in the study and did not assess higher energies. Higher energies can provide even higher dose rates (i.e., 2400 MU/min maximum dose rate for 10X‐FFF), thus one can expect further reduction of beam‐on time compared with their flattened counterparts. Nevertheless, in our study we investigated the most common application of DIBH left breast irradiation with the most commonly used beam energy. In addition, the improved target homogeneity achieved by the ECOMP technique has also reduced the need of high or mixed energy beams in patients with larger breasts that conventionally required such energies. Based on these results, we also postulate that FFF ECOMP planning technique could be used for other sites where forward treatment planning is typically used and reduced delivery time might have a big impact; for example, when the patient is in excruciating pain.

## 5 Conclusion

Our results demonstrate that ECOMP planning with 6X‐FFF is feasible for left‐sided whole breast irradiation with DIBH. With good delivery accuracy, 6X‐FFF ECOMP plans can significantly reduce the beam‐on time and hence required breath‐hold lengths compared with 6X plans. Dosimetrically, 6X‐FFF ECOMP plans can provide comparable target coverage and improved OAR doses to the contralateral breast and skin.

## Conflict of Interest

No conflict of interest.
